# Adjunctive therapy with lipid-lowering agents in COVID-19: a systematic review and meta-analysis of randomized controlled trials

**DOI:** 10.1186/s12944-023-01828-w

**Published:** 2023-05-08

**Authors:** Amirmohammad Khalaji, Amir Hossein Behnoush, Sanam Alilou, Malihe Rezaee, Soheil Peiman, Amirhossein Sahebkar

**Affiliations:** 1 Tehran Heart Center, Cardiovascular Diseases Research Institute, Tehran University of Medical Sciences, Tehran, Iran; 2 School of Medicine, Tehran University of Medical Sciences, Tehran, Iran; 3 Non–Communicable Diseases Research Center, Endocrinology and Metabolism Population Sciences Institute, Tehran University of Medical Sciences, Tehran, Iran; 4 School of Medicine, Iran University of Medical Sciences, Tehran, Iran; 5 Department of Pharmacology, School of Medicine, Shahid Beheshti University of Medical Sciences, Tehran, Iran; 6 Department of Internal Medicine, AdventHealth Orlando Hospital, Orlando, FL USA; 7 Biotechnology Research Center, Pharmaceutical Technology Institute, Mashhad University of Medical Sciences, Mashhad, Iran; 8 Applied Biomedical Research Center, Mashhad University of Medical Sciences, Mashhad, Iran; 9 School of Medicine, The University of Western Australia, Perth, Australia; 10 Department of Biotechnology, School of Pharmacy, Mashhad University of Medical Sciences, Mashhad, Iran

**Keywords:** COVID-19, Hydroxymethylglutaryl-CoA Reductase Inhibitors, Omega-3, Fibric Acid, Nicotinamide

## Abstract

**Background:**

Many commonly used drugs were evaluated as repurposed treatment options since the emergence of the COVID-19 pandemic. The benefit of lipid-lowering agents has been controversial in this regard. In this systematic review, we assessed the effect of these medications as adjunctive therapy in COVID-19 by the inclusion of randomized controlled trials (RCTs).

**Methods:**

We searched four international databases including PubMed, the Web of Science, Scopus, and Embase for RCTs in April 2023. The primary outcome was mortality, while other efficacy indices were considered secondary outcomes. In order to estimate the pooled effect size of the outcomes, considering the odds ratio (OR) or standardized mean difference (SMD) and 95% confidence interval (CI), random-effect meta-analyses was conducted.

**Results:**

Ten studies involving 2,167 COVID-19 patients using statins, omega-3 fatty acids, fenofibrate, PCSK9 inhibitors, and nicotinamide as intervention compared to control or placebo, were included. No significant difference was found in terms of mortality (OR 0.96, 95% CI 0.58 to 1.59, *p*-value = 0.86, *I*^*2*^ = 20.4%) or length of hospital stay (SMD -0.10, 95% CI -0.78 to 0.59, *p*-value = 0.78, *I*^*2*^ = 92.4%) by adding a statin to the standard of care. The trend was similar for fenofibrate and nicotinamide. PCSK9 inhibition, however, led to decreased mortality and an overall better prognosis. Omega-3 supplementation showed contradicting results in two trials, suggesting the need for further evaluation.

**Conclusion:**

Although some observational studies found improved outcomes in patients using lipid-lowering agents, our study found no benefit in adding statins, fenofibrate, or nicotinamide to COVID-19 treatment. On the other hand, PCSK9 inhibitors can be a good candidate for further assessment. Finally, there are major limitations in the use of omega-3 supplements in treating COVID-19 and more trials are warranted to evaluate this efficacy.

**Supplementary Information:**

The online version contains supplementary material available at 10.1186/s12944-023-01828-w.

## Introduction

With the emergence of the coronavirus disease 2019 (COVID-19) pandemic worldwide, about 650 million people were affected and about 6.5 million died as of December 9, 2022 [1]. The clinical manifestation of COVID-19 encompasses a wide range ranging from mild to severe symptoms, complications of which could be acute respiratory distress syndrome (ARDS) and end-organ failure resulting from inflammatory cytokine storm [2, 3]. Hitherto, there have been some drugs approved or authorized by the Food and Drug Administration (FDA) for treating COVID-19. In addition, some monoclonal antibodies and immunomodulatory agents have shown promising results in severe cases, but they are expensive and difficult to avail [4]. Therefore, a wise approach would be to identify the available drugs that could reduce the COVID-19 infection severity and improve the overall outcomes.

Previously, it has been demonstrated that patients with dyslipidemia are at higher risk of severe COVID-19 in addition to higher mortality [5]. Additionally, the reverse relation between 3-hydroxy-3-methylglutaryl Coenzyme A (HMG-CoA) reductase expression and the risk of COVID-19 hospitalization was found, indicating the possible benefit of statins in reducing the COVID-19 severity [6]. Moreover, as endothelial injury has been suggested to occur in COVID-19 [7], the protective role of statins for the endothelium might be effective as adjunctive treatment in COVID-19 [8]. The anti-inflammatory, anti-oxidative, and immunomodulatory properties of some lipid-modulating drugs such as omega-3 supplementations [9] and statins [10] could contribute to the lessening of inflammatory cytokine storm during severe COVID-19 infection. Moreover, in another pathway, the anti-inflammatory role of statins in cardiovascular diseases [11] might be a mediator in lessening the severity of COVID-19, since cardiovascular comorbidities and complications have been reported as essential contributors to the poor prognosis of COVID-19 [12]. In this regard, several randomized controlled trials (RCTs) identified the effects of lipid-modulating medications such as omega-3 supplementation [13], statins [14], nicotinamide [15], and fenofibrate [16] on the outcomes and severity of COVID-19. The findings of these studies have not been fully conclusive, with some demonstrating benefit in improving the outcomes [13–15], while others failing to find significant effects [16]. Discrepant results also exist with respect to previous meta-analyses [17], as these included observational studies that are usually accompanied by uncontrolled confounding bias. Therefore, we aimed to conduct a meta-analysis of conducted trials on the possible effects of lipid-lowering medications on the outcomes of COVID-19 patients, which can be helpful in the management of the patients.

## Methods

### Search strategy

PRISMA (Preferred Reporting System for Systematic Reviews and Meta-analyses) was used as the guideline for the conduction of this systematic review [18]. Databases including PubMed, Web of Science, Embase, and Scopus were investigated for trials evaluating the efficacy and/or safety of lipid-lowering agents as an adjunctive medical treatment for COVID-19 from inception to April 2023. Two main groups of keywords were used in the search: 1) lipid-lowering agents (statins, omega-3 fatty acid supplements, Fibrates, bile acid sequestrant, nicotinic acid, PCSK9 inhibitors, adenosine triphosphate citrate synthase inhibitors, and cholesterol absorption inhibitors), and 2) COVID-19. Details of searched keywords are available in Supplementary Table 1. No limitations or filters were added to the search query. This systematic review was registered in PROSPERO (registration number: CRD42023415932).

### Study selection, screening, and data extraction

We included RCTs comparing outcomes between COVID-19 patients receiving lipid-lowering agents (intervention group) and placebo (control group). Mortality was the primary outcome, while the secondary outcomes were mechanical ventilation need, hospital and/or intensive care unit (ICU) length of stay, bleeding, clinical deterioration (defined as ﻿WHO Ordinal Scale ≥ 6 [19] *i.e.*, non-invasive ventilation, the requirement for high flow oxygen, administration of vasopressor agents, endotracheal intubation, renal replacement therapy, mortality, and extracorporeal membrane oxygenator (ECMO) requirement), venous thromboembolism, and shock. We excluded case series, case reports, observational studies, conference abstracts, and non-English articles.

At first, duplicates were excluded. Then, according to the title and abstract, two independent reviewers (AK and AHB) initially included all studies that were related to adding lipid-lowering agents to the treatment plan of COVID-19 patients. Then, using the full text of the article, RCTs that compared the outcomes between the lipid-lowering group and the control group were included. The third reviewer (SA) resolved disagreements between the two reviewers.

After the screening, two reviewers (AK and AHB) performed data extraction in a prespecified spreadsheet. The following data were extracted: 1) RCT name, first author’s name, and publication year, 2) RCT design, study population, intervention group, and control group, 3) numbers of the total population, intervention, and control groups, 4) mean age, male percentage, and main findings.

### Quality assessment

Qualities of included RCTs were evaluated using the Cochrane risk-of-bias tool for randomized trials (RoB-2) [20]. While two authors (AK and AHB) performed the risk of bias assessment independently, a third author (SA) was responsible for resolving any possible disagreement. In brief, RoB-2 criteria include five domains of bias source. These are the randomization process, missing outcome data, deviations from the intended interventions, measurement of the outcome, and selection of the reported result, all of which can be graded as “low-risk”, “high-risk” or “some concern”.

### Statistical analysis and data synthesis

Data were extracted as means (standard deviation, SD) or median [interquartile range, IQR] for the COVID-19 clinical outcomes and complications to find the difference between the intervention and control arms of the included trials. When possible, the random-effect meta-analysis was performed with the DerSimonian-Laird model to find the pooled effect size of the outcomes via calculation of the odds ratio (OR) along with a 95% confidence interval (CI). Also, regarding continuous outcomes such as length of hospital stay, we performed a random-effect meta-analysis to calculate the standardized mean difference (SMD) and 95% CI. When possible, subgroup analyses based on the dosage of medications were performed. For evaluation of heterogeneity, Higgins' I-square test was utilized with ranges of ≤ 25%, 26–75%, and > 75 in *I*^*2*^ considered as “low”, “moderate”, and “high” heterogeneity, respectively. The *p*-value of < 0.05 was considered a statistical significance cutoff. All analyses were performed with STATA software (Stata Corp, version 17).

## Results

### Included study characteristics

**Fig. 1:**
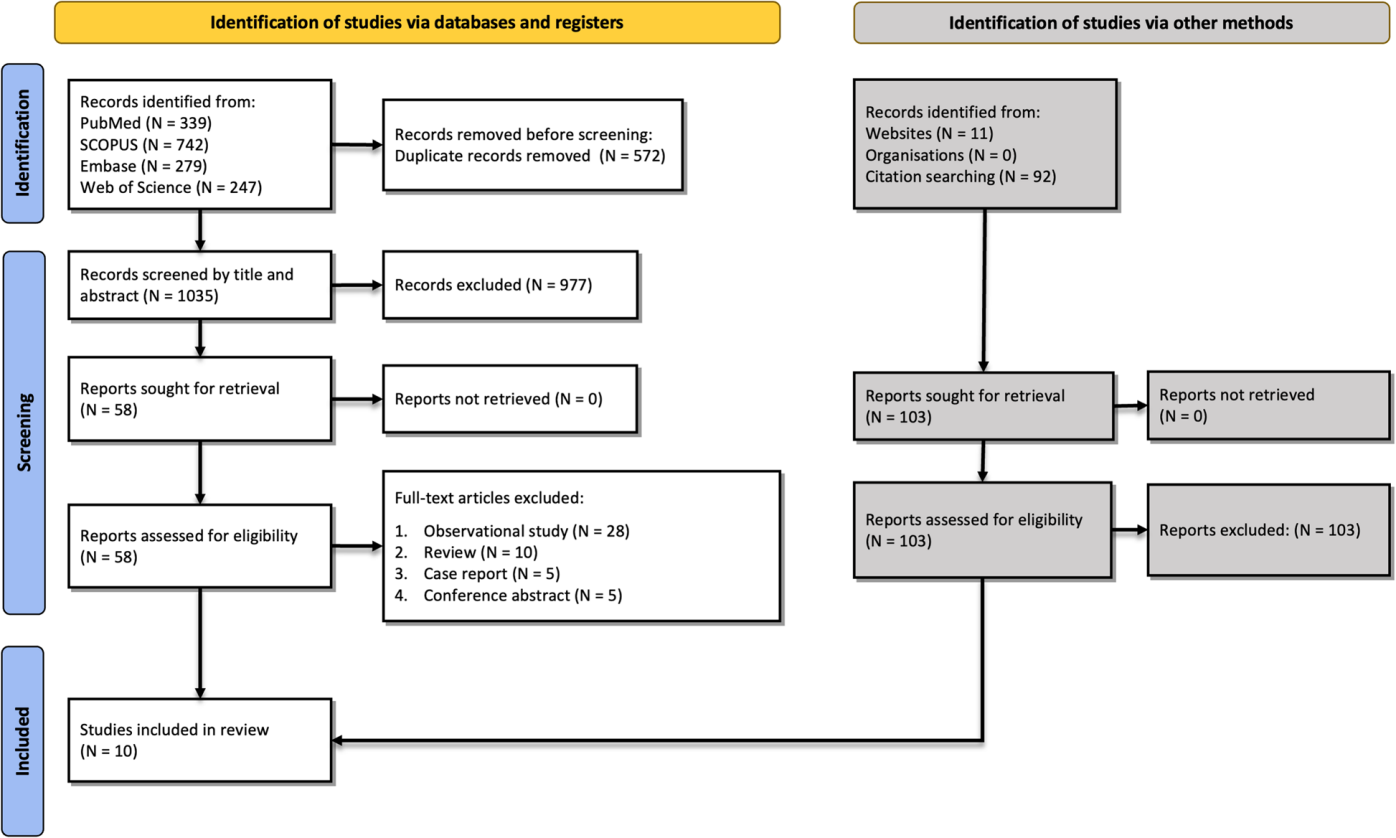
Flow diagram summarizing the selection of eligible studies based on the PRISMA guidelines

The initial search with the keywords explained in supplementary table 1 resulted in 1,607 studies. Finally, after removing duplicates and screening by title and abstract and then full-text of studies, 10 articles remained to be included in our study [13–16, 21–26]. A letter [27] was excluded from our systematic review due to the same population as the study by Pawelzik et al. [26]. All included studies evaluated the therapeutic role of lipid-lowering agents in COVID-19 patients. The search details and selection process are shown in Fig. 1.

**Fig. 2:**
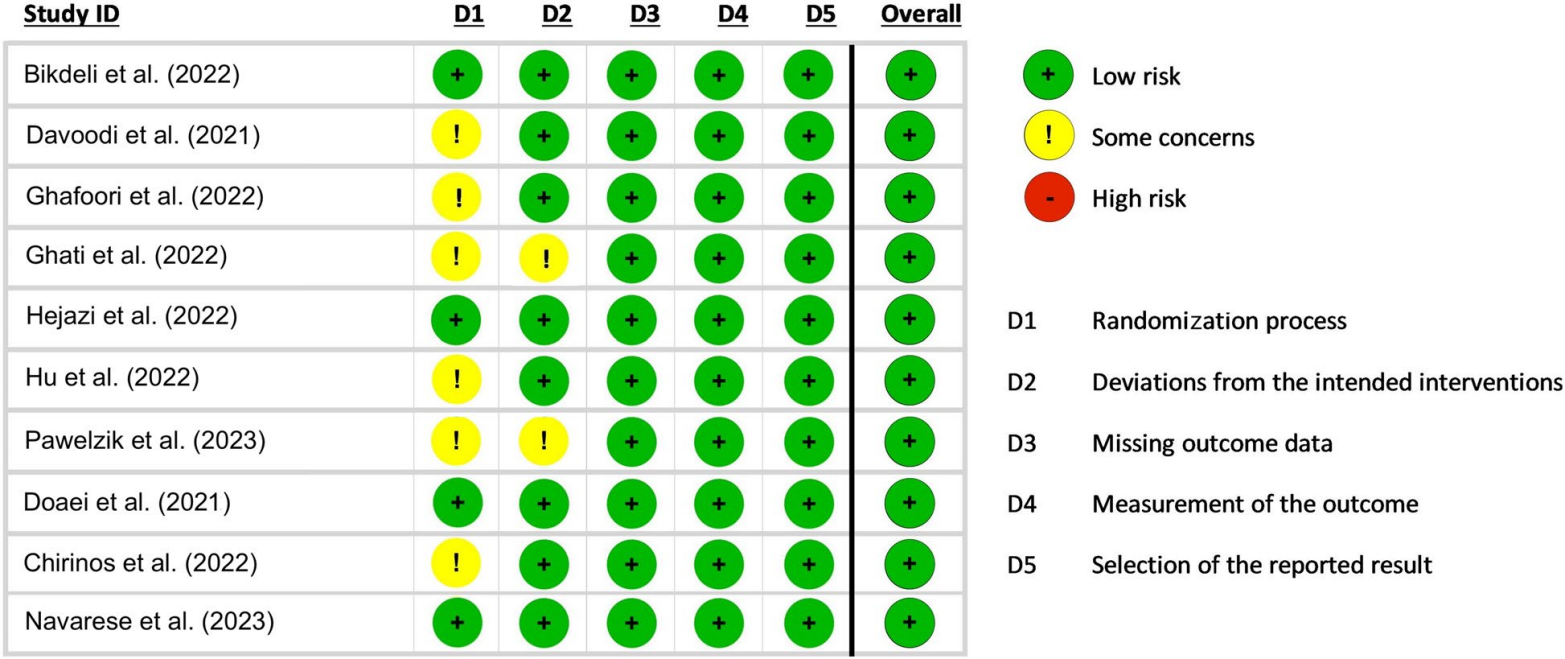
Quality assessment of the included trials based on the Cochrane Risk of Bias tool

The baseline features of the studies included in this review are shown in Table 1. Atorvastatin was assessed in five trials [14, 21–24], omega-3 in two [13, 26], nicotinamide in one [15], fenofibrate in one [16], and PCSK9 inhibitors in one [25]. Most of the studies included hospitalized patients, except for Bikdeli et al. [21] trial which was on COVID-19 cases admitted to ICU, Doaei et al. [13] al. and Navarese et al. [25] trials which included critically ill COVID-19 cases, and Chirinos et al. [16] study assessed both inpatients and outpatients. A total of 2,167 patients were included, among which 1,058 were randomized to anti-lipid medications, while 1,109 were in the control/placebo group. The patients’ mean age was 54.2 ± 14.9 years and 1,270 (58.6%) were male. Details of the risk of bias assessment of the included trials based on Cochrane’s tool are shown in Fig. 2. The first domain (randomization process) was the most frequent bias with “some concerns”.

**Table 1:** Baseline characteristics of the included studies

Author	Year	Design	Agent	Population	Intervention	Control	Age (years)	Male (%)	Main findings
Bikdeli et al. [21]	2022	2*2 factorial RCT	Statin	COVID-19 cases admitted to ICU (*n* = 587)	Atorvastatin 20 mg orally once daily (*n* = 290)	Placebo (*n* = 297)	56.4 ± 16.7	56.4	No significant difference in the occurrence of the primary outcome (arterial thrombosis, venous thrombosis, ECMO, or 30-day mortality) between the atorvastatin group (95 patients; 33%) and the placebo (108 patients; 36%) with an OR of 0.84 [95% CI 0.58–1.21]
Davoodi et al. [14]	2021	Double-blind RCT	Statin	Hospitalized COVID-19 cases (*n* = 40)	Atorvastatin 40 mg orally once + lopinavir/ritonavir (*n* = 20)	Lopinavir/ritonavir (*n* = 20)	46.0 ± 6.9	52.5	The hospital stay duration was significantly reduced in the lopinavir/ritonavir + atorvastatin group in comparison with the control group (P = 0.012). However, no significant difference was observed between the need for mechanical ventilation and the need for immunoglobulin and interferon
Ghafoori et al. [22]	2022	Open-label RCT	Statin	Hospitalized COVID-19 cases (*n* = 154)	Atorvastatin 20 mg orally once daily + lopinavir/ritonavir (*n* = 76)	Lopinavir/ritonavir (*n* = 78)	50.6 ± 21.1	50.6	A total of seven patients died, including two patients (2.6%) from controls and five (6.6%) in the atorvastatin group. The mean hospitalization duration days (p = 0.001) and the frequency of hospitalization in the ICU ward (18.4% vs. 1.3%) were longer in the intervention group. Moreover, the pulse rate (p = 0.004) was reported to be higher in the intervention group
Ghati et al. [23]	2022	Open-label RCT	Statin	Hospitalized COVID-19 cases (*n* = 440)	Atorvastatin 40 mg orally once daily (*n* = 221)	Control (*n* = 219)	52.2 ± 10.4	73.7	There was no statistical difference between the atorvastatin and the control groups in terms of mortality, mechanical ventilation, clinical deterioration, and hospital stay length
Hejazi et al. [24]	2022	Triple-blind RCT	Statin	Hospitalized COVID-19 cases (*n* = 40)	Atorvastatin 20 mg orally once daily (*n* = 20)	Placebo (*n* = 20)	54.6 ± 14.7	70.0	Atorvastatin had a significant impact on the reduction of oxygen need, serum hs-CRP levels, and hospitalization duration in hospitalized COVID-19 patients with mild-to-moderate disease
Doaei et al. [13]	2021	Double-blind RCT	Omega-3	Critically ill COVID-19 patients (*n* = 101)	Omega-3 1000 mg daily (*n* = 28)	Nutritional support (*n* = 73)	64.5 ± 14.3	59.4	The one-month survival rate was significantly higher in the intervention group. Also, higher levels of arterial pH, HCO3, and Be and lower levels of BUN, Cr, and K were found in the intervention group compared with the control group (all p < 0.05)
Pawelzik et al. [26]	2023	Open-label RCT	Omega-3	Hospitalized COVID-19 cases (*n* = 20)	n-3 PUFA emulsion containing 0.1 g/mL of fish oil (*n* = 10)	Placebo (*n* = 10)	80.7 ± 6.2	45	IV n-3 PUFA changed eicosanoid metabolites and decreased inflammatory and thrombosis mediators’ levels. Moreover, 15-F_2t_-isoprostane, as an oxidative stress marker was reduced in the intervention arm who had lower erythrocyte oxidative stress as well
Chirinos et al. [16]	2022	Double-blind RCT	Fenofibrate	COVID-19 cases (outpatient and inpatient) (*n* = 701)	Fenofibrate (*n* = 351)	Placebo (*n* = 350)	49 ± 16	52.9	There was no statistical difference in all-cause mortality between the arms. There were 61 (17%) adverse events reported in the placebo arm in comparison to 46 (13%) in the fenofibrate group. Additionally, the incidence of gastrointestinal side effects was slightly higher in patients receiving fenofibrate
Hu et al. [15]	2022	Open-label RCT	Nicotinamide	Hospitalized COVID-19 cases (mild/moderate) (*n* = 24)	Nicotinamide (*n* = 12)	Routine treatments (*n* = 12)	69.5 ± 12	45.8	In COVID-19 patients, the whole blood counts and absolute lymphocyte counts did not change significantly in any of the groups (intervention and control) (*p* > 0.05)
Navarese et al. [25]	2023	Double-blind RCT	PSCK9 inhibitor	Severe hospitalized COVID-19 patients (*n* = 60)	Evolocumab (*n* = 30)	Placebo (*n* = 30)	66.1 ± 12	61.7	Patients receiving PCSK9 inhibitor exhibited a lower rate of the primary endpoint (30-day mortality or need for intubation) (23.3% vs. 53.3%). Also, the intervention group had significantly lower oxygen therapy duration and length of hospital stay, compared to the placebo group

Data are presented as mean ± standard deviation or percentage

*RCT* Randomized controlled trial, *ICU* intensive care unit, *CI* confidence interval, *PUFA* polyunsaturated fatty acid, *PCSK9* Proprotein convertase subtilisin/kexin type 9

### Statins and COVID-19

#### Mortality

**Fig. 3:**
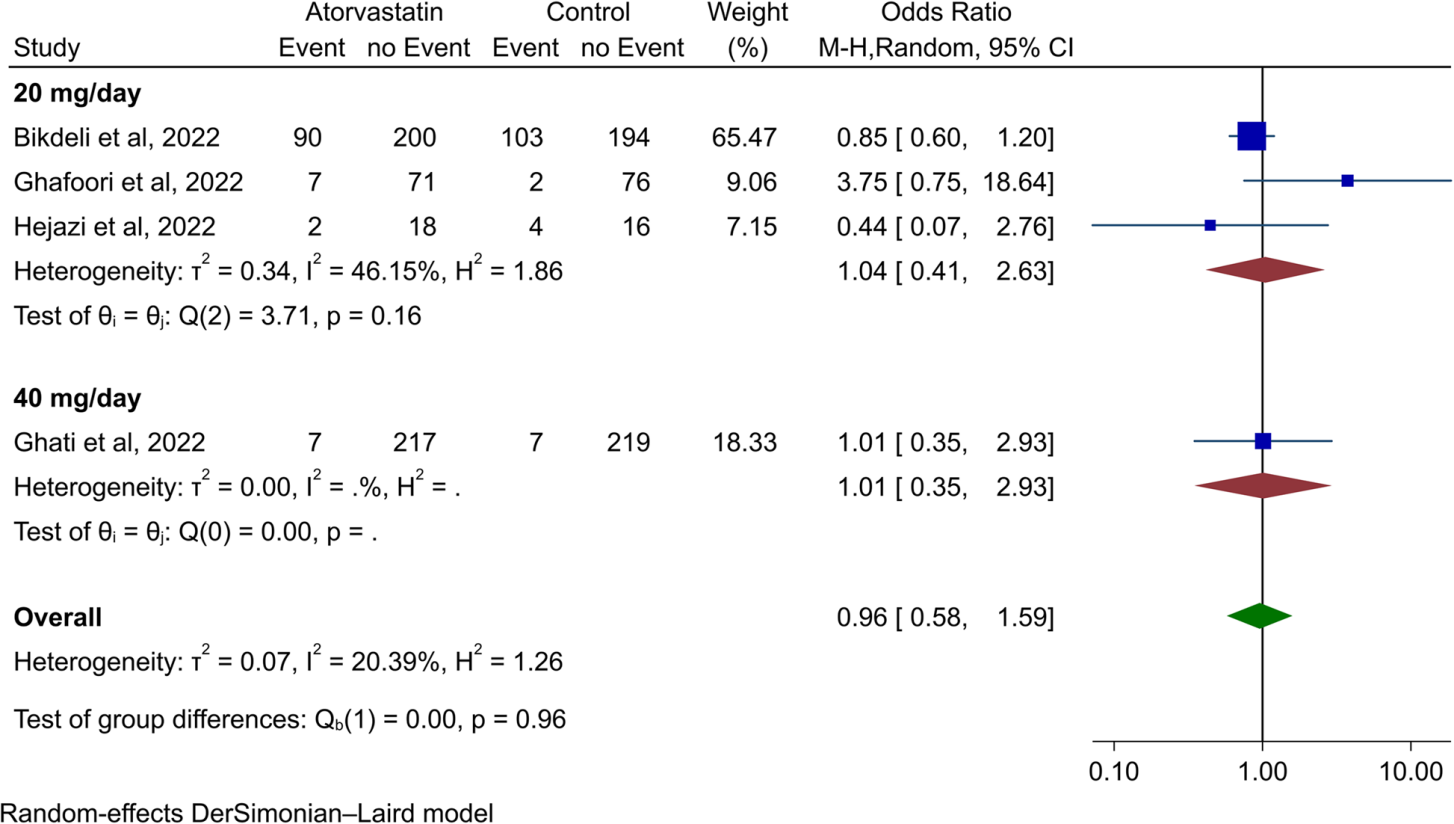
Forest plot for the meta-analysis of mortality for statin therapy and subgroup analysis based on atorvastatin dosage

Meta-analysis was performed to estimate the effect of atorvastatin as added treatment in reducing mortality in four studies. The result showed no statistically significant difference (OR 0.96, 95% CI 0.58 to 1.59, *p*-value = 0.86, Fig. 3). While the meta-analysis had a mild degree of heterogeneity (*I*^2^: 20.39%), no significant difference was observed in every single study. Also, in the subgroup analysis based on statin dosage, no difference in mortality was observed in any of the 20 mg/day and 40 mg/day dosages (Fig. 3). While there was no significant difference between the two subgroups (*p*-value = 0.96), also there was no significant difference in mortality between statin and placebo groups in the 20 mg/day subgroup analysis (OR 1.04, 95% CI 0.41 to 2.63, *p*-value = 0.93, Fig. 3). In addition to the fact that none of the individual studies reported significant differences between the groups, pooling their results also led to the same insignificant difference.

#### Mechanical ventilation

Two of the studies investigated the need for ventilation among COVID-19 patients by randomizing them into intervention and control arms [14, 23]. Neither of these two studies showed any significant difference between placebo and statins. Davoodi et al. reported 0/20 in the intervention arm and 1/20 in the control group (*p*-value > 0.05). Similarly, Ghati et al. found an insignificant difference between the groups (7/224 in the intervention group and 6/226 in the control arm).

#### Hospital length of stay

**Fig. 4:**
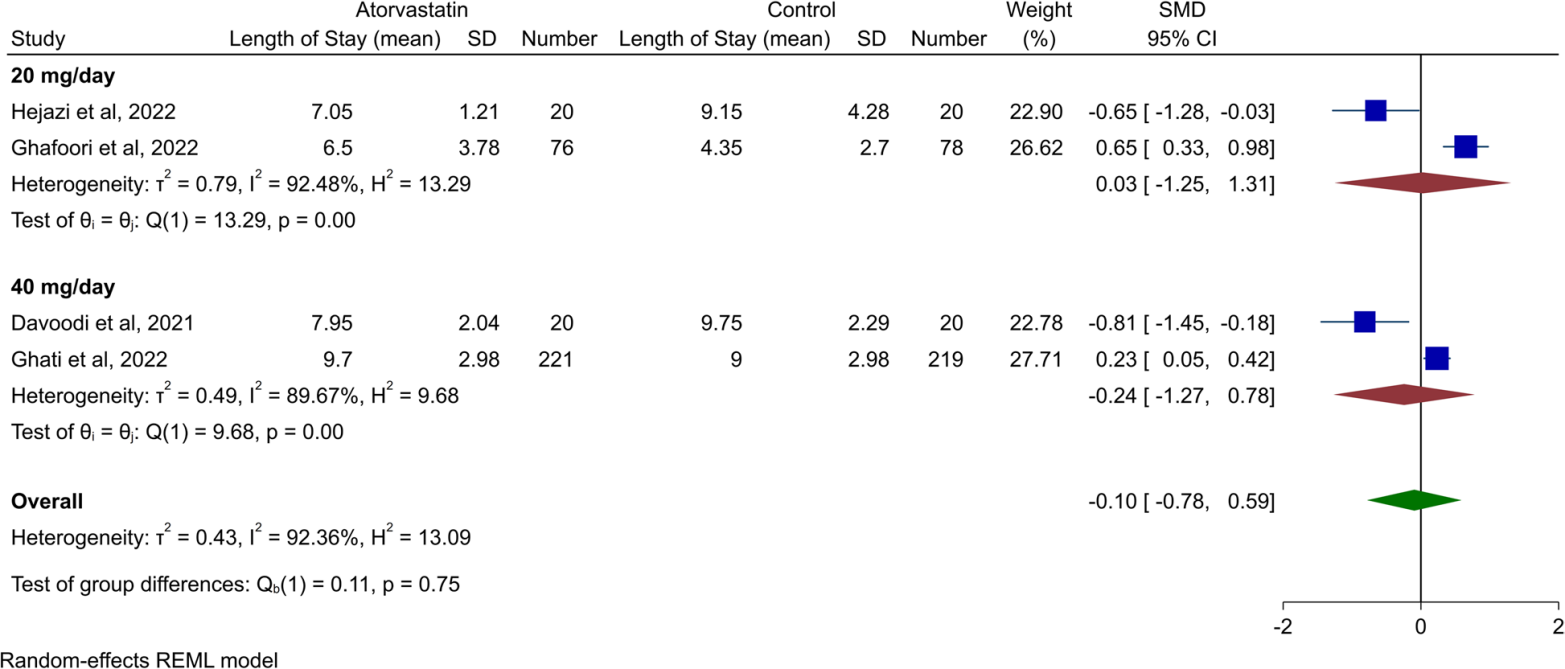
Forest plot for the meta-analysis of the length of hospital stay for statin therapy and subgroup analysis based on atorvastatin dosage

The duration of hospitalization stay was assessed in four of the studies. Davoodi et al. found a significantly shorter hospitalization duration in comparison with the control arm (7.95 ± 2.04 days vs. 9.75 ± 2.29 days, *p*-value = 0.012). In line, Hejazi et al. came to the same conclusion (7.05 ± 1.21 days vs. 9.15 ± 4.28 days, *p*-value = 0.03). On the other hand, Ghafoori et al. reported a significantly longer length of stay in comparison with controls (6.5 [4-9] days vs. 4 [3-6] days, *p*-value = 0.001). Ghati et al. reported an almost identical length of stay for the groups with atorvastatin and the control arm (9 [8-12] days vs. 9 [7-11] days, *p*-value = 0.85). Meta-analysis of hospital length of stay from these four studies revealed that there is no significant difference between patients randomizing to atorvastatin in comparison with the control group (SMD -0.10, 95% CI -0.78 to 0.59, *p*-value = 0.78, Fig. 4). In subgroup analysis based on dosage, none of the 20 mg and 40 mg daily dosages showed any statistical difference in terms of hospitalization days.

Finally, the duration of ICU stay was also measured in Bikdeli et al. trial, in which there was no significant difference between the atorvastatin and placebo groups (5 [3-9] days vs. 5 [2-10] days, *p*-value = 0.31).

#### Other complications

Other complications were evaluated in studies. Venous thromboembolism was reported by Bikdeli et al. not to be significantly different between the arms (*p*-value = 0.53). This study could not show any significant difference in terms of bleeding events (*p*-value > 0.05), either. Ghati et al. did not report any significant difference in clinical deterioration and shock between atorvastatin and control groups (*p*-value > 0.05 for both outcomes).

#### Omega-3 fatty acid supplementation and COVID-19

Two studies evaluated the efficacy of supplementation with omega-3 fatty acids in COVID-19 patients [13, 26]. Doaei et al. [13] found a significantly higher 30-day survival rate in the intervention group (omega-3 1000 mg daily) compared with the control, which received only nutritional support (21% vs. 3%, *p*-value = 0.003) in a double-blind RCT of ICU-admitted COVID-19 patients. Moreover, omega-3 fatty acids significantly improved kidney function, assessed through creatinine levels (1.29 vs. 1.68 mg/dL, *p*-value = 0.02), in the intervention group. However, omega-3 supplementation could not increase Glasgow Coma Scale (GCS) and lymphocyte count in the intervention arm in a significant manner (*p*-value > 0.05).

In another single-blind trial by Pawelzik et al. [26] on hospitalized COVID-19 patients, it was shown that intravenous n-3 polyunsaturated fatty acid (PUFA) significantly decreased the levels of thrombosis and inflammation mediators, while it increased the prostacyclin levels (*p*-value < 0.05). Moreover, 15-F_2t_-isoprostane, as an oxidative stress marker, was decreased significantly in the intervention arm, compared with patients receiving a placebo. Finally, reactive oxygen species were lower in the erythrocytes of COVID-19 patients receiving n-3 PUFA.

#### Fibrates and COVID-19

One double-blind RCT investigated the efficacy of fenofibrate *versus* placebo in COVID-19 patients [16]. No significant difference in efficacy measures was found between the fenofibrate and placebo groups. The studied groups were comparable in terms of the global ranked severity score without any significant difference (5.32 [2.98—6.00] *vs.* 5.33 [2.98—6.00], *p*-value = 0.819), the number of days alive, out of ICU/ECMO/invasive ventilation (30 [30-30] *vs.* 30 [30-30], *p*-value = 0.134), WHO ordinal scale (1 [1-1] *vs.* 1 [1-2], *p*-value = 0.246), and modified ranked severity score (5.05 [2.98—5.22] *vs.* 5.05 [2.98—5.21], *p*-value = 0.928).

#### Nicotinamide and COVID-19

Nicotinamide was another lipid-lowering agent assessed by Hu et al. [15] in an open-label RCT. The intervention group included COVID-19 patients with lymphopenia receiving 100 mg nicotinamide five times a day for two days, while the control arm received usual care. No significant difference was detected between the two groups in laboratory assessments, including absolute lymphocyte count (*p*-value = 0.67), C-reactive protein (CRP) (*p*-value = 0.76), and other full blood counts (*p*-value > 0.05).

#### PSCK9 inhibitor and COVID-19

The study by Navarese et al. assessed the impact of PCSK9 inhibition in the severe COVID-19 [25]. In this double-blind RCT, patients receiving evolocumab showed a significantly lower death rate or the need for intubation (23.3% vs. 53.3%, risk difference of -30% (95% CI -53.40% to -6.59%), *p*-value < 0.05). Moreover, interleukin-6 levels were lower in COVID-19 patients randomized to PCSK9 inhibitors.

## Discussion

This study investigated the efficacy of anti-dyslipidemic agents in improving COVID-19 outcomes through a systematic review and meta-analysis of RCTs. The main findings of the current study can be summarized as 1) Adding statins to the standard of care did not show any beneficiary effect in improving COVID-19 outcomes, 2) omega-3 fatty acid supplementation resulted in conflicting outcomes which need further evaluation, 3) in only one trial, fibrates had no significant effect on COVID-19 severity, 4) nicotinamide did not affect lymphocyte count in lymphopenic patients with COVID-19, and 5) PCSK9 inhibitors (evolocumab) reduced death rate or the need for intubation in COVID-19 patients. While there was a larger population included in statin trials, other anti-dyslipidemic had smaller studied populations which necessitate further studies to be done.

Since the efficacy of antiviral drugs and corticosteroids in treating COVID-19 is not well-proven [28], studies determined the effectiveness of more available and cheaper medications such as statins [29], antiplatelets [30], and anticoagulants [31] in decreasing post-infection complications. The rationale behind choosing anti-dyslipidemic agents (*e.g.,* statins) in COVID-19 patients is the role of dyslipidemia and cardiovascular complications in the prognosis of COVID-19. Studies have shown a meaningful association between dyslipidemia and the severity of COVID-19 in addition to mortality in COVID-19 patients [32]. Statins decrease the cholesterol level by inhibiting HMGCR and studies in COVID-19 patients have suggested a potential relationship between inhibiting HMGCR and a lower rate of hospitalization [6].

The results of five trials assessing the effectiveness of statins in COVID-19 have been reported so far. Statins are the first line of treatment for dyslipidemia and their effectiveness in improving lipid profile has been well-proven in the past [33]. In addition to their lipid-lowering effects, previous studies found statins to be potentially beneficial in preventing and treating infections [34]. In a retrospective cohort, chronic use of statins was correlated with lower one-year mortality in respiratory viral infection [35]. At the beginning of the COVID-19 pandemic, several studies explored the relationship between using statins and preventing or treating COVID-19. Moreover, several meta-analyses have been conducted in the investigation of statin efficacy in COVID-19 patients which are summarized and described in Table 2 and Supplementary Materials. There were conflicting results found in these studies which were predictable due to the observational nature of included studies in all of these systematic review studies. Additionally, the efficacy of adding statins to treatment regimens of COVID-19 patients remained controversial. Interestingly and in contrast with observational studies, none of our included studies found significant differences in terms of mortality. However, their pooled results by meta-analysis also revealed no difference. The rationale for performing a meta-analysis of mortality despite all these insignificant results was to provide stronger evidence for literature against observational studies and meta-analyses with the inclusion of these studies. This can clarify the fact that adding statins to the normal regimen of COVID-19 patients have no added value, based on the pooled result of randomized trials.

**Table 2:** Summary of the results of previous meta-analyses on observational studies

	**Author, Year**	**Included studies design**	**Number of included studies**	**Main Findings**
1	Lao et al., 2022	Retrospective observational which reported covariate adjusted effect estimates	70	Statin was associated with reduced mortality, ICU admission, and mechanical ventilation
2	Vahedian-Azimi et al., 2021	Observational studies	23	Statin use had no significant effect on ICU admission and all-cause mortality, however, it decreased need for intubation
3	Diaz-Arocutipa et al., 2021	Cohort studies	25	Unadjusted risk ratio meta-analysis did not show a reduction in mortality, while adjusted odds and hazard ratio meta-analysis resulted in significant association of statins and mortality reduction
4	Kow et al., 2021	Original observational studies and preprints	35	Use of statins was associated with a significantly lower risk of all-cause mortality and endpoint of severe illness
5	Yetmar et al., 2021	Cohorts and case–control studies (observational)	19	Prior statin use was associated with a lower risk of mortality and severe COVID-19
6	Kollias et al., 2021	Prospective or retrospective design	22	Statin use was associated with lower risk of mortality from COVID-19
7	Zein et al., 2022	Propensity-matched cohorts	8	Decreased mortality was observed to be associated with statin use
8	Chow et al., 2021	Cohort studies	13	Risk of mortality was similar between statin users and non-users. However, the patients started their statin use after COVID-19 diagnosis had lower mortality
9	Wu et al., 2021	Observational studies	28	Use of statins was significantly associated with decreased mortality and invasive mechanical ventilator support
10	Vahedian-Azimi, 2021	Observational studies	24	Significant reduction was observed in statin users in terms of ICU admission, and death. However, no difference was observed in tracheal intubation
11	Hariyanto et al., 2021	Observational studies	35	Statin use did not improve the composite poor outcomes of COVID-19
12	Permana et al., 2021	Retrospective cohort	13	In-hospital statin use was associated with reduced risk of mortality, while pre-admission use of statins was not related to mortality
13	Pal et al., 2022	Observational studies	14	Unadjusted data meta-analysis revealed no improved clinical outcomes, while adjusted risk estimated showed significant reduction in adverse outcomes of COVID-19
14	Hariyanto et al., 2020	Observational studies	9	Statin use did not affect in-hospital outcomes of COVID-19

Full reference citation of these meta-analyses are in Supplementary Materials; ICU: intensive care unit

To our knowledge, ours is the first systematic review of RCTs comparing the efficacy of statins as an adjuvant medication in the COVID-19 treatment plan. Two of the included studies reported recent statin use as an exclusion criterion [14, 23]. We found no benefit in adding statins for reducing mortality, need for mechanical ventilation, or duration of hospital stay. Contrary to our findings, a recent systematic review and meta-analysis of 84 observational studies found that statin use significantly lowers mortality, ICU admission, and the need for mechanical ventilation in COVID-19 patients [17]. Especially in the case of statins, observational studies are prone to several biases and have many confounders that could affect the results. The lack of a placebo group is the most important limitation of observational studies on COVID-19. Secondly, the dosage of statins is not constant in observational studies and can cause bias in the interpretation of results. According to our findings, the results of previous studies cannot be confirmed by RCTs and statins have no discrete role in the treatment plan of COVID-19 patients. However, statin use in patients with dyslipidemia may improve COVID-19 outcomes, which highlights the need for trials including COVID-19 in patients with dyslipidemia. It should be noted that despite these results observed in our meta-analysis, more studies can be conducted to confirm these findings for not wasting resources in the prescription of statins. Finally, as our results do not include patients with current cardiovascular disease or the ones at higher risk infecting with COVID-19, statins should not be removed from their regimens.

Regarding the length of hospital stay, the statins had conflicting results among different trials for which our meta-analysis found overall no significant difference between statins and control. However, it should be noted that there are several factors influencing COVID-19’s length of hospital stay. These include but are not limited to age, the seriousness of the illness (breathing difficulty, organ failure, and leukopenia), patient-to-healthcare workers ratio, and treatment outcome at discharge in addition to other factors such as geographic location [36, 37]. Thus, there are serious limitations in drawing a conclusion about the effect of statins on hospitalization length.

The second lipid-lowering drug that was investigated in two trials is omega-3. Supplementation with omega-3 reduces systemic inflammation in SARS-CoV-2 infection by reducing the CRP levels [38]. Moreover, blood levels of omega-3 were lower in more severe forms of COVID-19 [39]. Zapata et al. [40] found a lower omega-3 index in COVID-19 patients compared to healthy controls. In addition, a significant negative association was detected between the omega-3 index and the need for mechanical ventilation and death in severe COVID-19 patients. To confirm the results of observational studies, RCTs by Doaei et al. [41] and Arnardottir et al. [27] investigated the efficacy of omega-3 in COVID-19 patients in terms of clinical outcomes and laboratory findings, respectively. Although Doaei et al. [41] reported better mortality and ICU admission outcomes. However, for omega-3 there is a long way to confirm the efficacy in the treatment of COVID-19. First, United States FDA has not approved omega-3 fatty acid dietary supplements as over-the-counter drugs [42], which severely limits their application and clinical use both as an anti-lipid agent and a candidate for COVID-19 treatment. Second, as there are two trials with contradicting results and without clear efficacy in them, future studies are needed to assess this role in managing COVID-19.

One of the main pathways of SARS-CoV-2 complications such as multiorgan failure is the inflammatory response caused by cytokine generation. Among them, interleukin-6 can predict COVID-19 severity, intubation risk, and mortality [43, 44]. In this regard, PCSK9 has been shown to increase inflammatory response and increased mortality in animal models, specifically, expression of interleukin-6 [45]. In the trial by Navarese et al. [25] also survival benefits of PCSK9 inhibition were observed especially in patients with a higher degree of inflammation. It seems that these agents can have direct inhibiting effects on inflammatory cascade and their role in lipid-lowering therapy.

Fibrates act as a lipid-lowering agent by inducing lipoprotein lipolysis and hepatic fatty acid uptake, reducing hepatic triglyceride production, increasing removal of low-density lipoprotein particles, increasing high-density lipoprotein production, and stimulating reverse cholesterol transport [46]. Previously, fenofibrate, a fibrate used in treating COVID-19, had shown anti-inflammatory effects in several diseases [47, 48]. Moreover, the effects of fenofibrate on the angiotensin-converting enzyme II receptors increased the possibility of the effectiveness of this fibrate in preventing viral entry and/or reducing COVID-19 severity [49]. Thus, an RCT tried to investigate the efficacy of fenofibrate in improving clinical outcomes in the COVID-19 [16]. Although previous studies proposed fenofibrate as an effective medication for COVID-19, Chirinos et al. [16] found no benefit in adding this drug to patients’ treatment regimens. All in all, more trials are needed to elucidate fibrates' role in preventing and treating COVID-19.

Nicotinamide as a water-soluble compound is metabolized by the liver and has renal excretion [50]. Its role in glycolysis has also been demonstrated, in which it generates Nicotinamide adenine dinucleotide (NAD^+^) for adenosine triphosphate production [51]. Lymphopenia in COVID-19 has been attributed to lymphocytic infiltration to target organs such as the lungs [52]; however, in the later stages, hyperinflammation, and release of cytokines is a key player [53]. The involvement of nicotinamide in COVID-19 pathogenesis has been suggested to be mediated in several ways. Nicotinamide-derived NAD^+^ modulates cytokine actions and intercellular adhesion molecules, inhibits mast cell degranulation, and blocks leukocyte protease release [54]. Moreover, the role of NAD^+^ metabolism by the enzyme CD38 has been suggested in COVID-19 [55]. Despite these clues, the only RCT that investigated the drug’s impact on COVID-19 lymphocyte count did not find any significant effect of adding nicotinamide to standard treatment [15]. Obviously, further studies are warranted to confirm these findings.

### Strengths and limitations

This study was the first to explore the role of anti-lipid agents as adjunctive agents in the treatment plan of COVID-19 patients through RCTs only. The inclusion of RCTs as the design with a lower risk of bias and confounders is the main strength of our study. However, the current study has several limitations deserving acknowledgment. First, there were relatively few trials for drugs other than statins to conclude their efficacy in COVID-19. Therefore, future multicenter RCTs with larger sample sizes are needed to provide more solid evidence. Second, there were some open-label trials in our included studies, which may pose the caveat of selection and performance biases in these studies, and consequently the pooled estimate. Third, the conducted studies fell short of including or subgrouping patients at a high cardiovascular risk, who may benefit more from statin therapy. Fourth, in the analysis of mortality, there were three studies with statin use of 20 mg daily, while one administered 40 mg of atorvastatin. Although we performed subgroup analysis, the low number of studies and this different dosage might confine our results. In addition to mortality analysis, the low number of studies investigating the need for mechanical ventilation that prohibits us from performing meta-analysis should be taken into consideration when interpreting the results. Finally, since there was heterogeneity between the RCTs in the reporting of the outcomes, most of the analyzed outcomes’ results were supported by a few trials, limiting the power and generalizability of the findings.

## Conclusion

In summary, the present meta-analysis of RCTs did not suggest a clear benefit of adding anti-dyslipidemic agents, in particular statins, to standard-of-care COVID-19 treatments. Current evidence does not support the benefit of de novo statin therapy in patients suffering from COVID-19. Concerning omega-3 fatty acids, the extant evidence from RCTs is still scant, and more and larger studies are needed to assess any causal effect on COVID-19 mortality. Our study’s findings can be used in clinical settings and prevent the loss of resources on the prescription of ineffective drugs for COVID-19. However, future studies such as large RCTs might be needed in some instances with lower levels of evidence.

## Supplementary Information

**Additional file 1: Supplementary Table 1.** Search strategy for each database.

## Data Availability

All data generated or analyzed during this study are included in this published article/as supplementary information.
