# Supplementary material for: Adjunctive therapy with lipid-lowering agents in COVID-19: a systematic review and meta-analysis of randomized controlled trials
Source: Lipids Health Dis. 2023 May 8;22:61. doi: 10.1186/s12944-023-01828-w (PMC10165571; doi:10.1186/s12944-023-01828-w)
Supplement: Supplementary file 1 — Additional file 1: Supplementary Table 1. Search strategy for each database. [file 12944_2023_1828_MOESM1_ESM.docx]

***Supplementary Materials***

**Adjunctive Therapy with Lipid-Lowering Agents in COVID-19: A Systematic Review and Meta-Analysis of Randomized Controlled Trials**

***Supplementary Table 1.*** *Search strategy for each database*

| **Query** | | **Results** |
| --- | --- | --- |
| ***PubMed*** | | |
| #1 | ("Statin*" OR Atorvastatin OR Fluvastatin OR Lovastatin OR Pitavastatin OR Pravastatin OR Rosuvastatin OR Simvastatin OR Cerivastatin OR "PCSK9 inhibitor*" OR "PCSK9" OR Evolocumab OR Alirocumab OR Inclisiran OR "Fibric acid*" OR "Fibrate*" OR Fenofibric OR Fenofibrate OR gemfibrozil OR "Bile acid sequestrant*" OR "bile acid resin*" OR Colesevelam OR Cholestyramine OR Colestipol OR "Nicotinic acid*" OR "Niacin" OR Nicotinic OR "pyridine-3-carboxylic acid" OR nicotinamide OR niacinamide OR niacin OR "pyridine-3-carboxamide" OR "vitamin B3" OR "Cholesterol absorption inhibitor*" OR Ezetimibe OR "Omega-3 fatty acid*" OR "Omega-3" OR "omega-3-acid" OR Lovaza OR "Adenosine triphosphate citrate synthase inhibitor*" OR "ATP citrate synthase inhibitor*" OR "Adenosine triphosphate-citrate synthase inhibitor*" OR "ATP-citrate synthase inhibitor*" OR "Adenosine triphosphate citrate-synthase inhibitor*" OR "ATP citrate-synthase inhibitor*" OR "ATP citrate lyase" OR Bempedoic) | 202,596 |
| #2 | ("COVID-19" OR "coronavirus" OR "SARS-CoV-2" OR "severe acute respiratory syndrome") | 366,858 |
| #3 | (trial OR random* OR intervention* OR placebo) | 3,776,812 |
| **#4** | **#1 AND #2 AND #3** | **339** |
| ***SCOPUS*** | | |
| #1 | (TITLE-ABS-KEY("Statin*" OR Atorvastatin OR Fluvastatin OR Lovastatin OR Pitavastatin OR Pravastatin OR Rosuvastatin OR Simvastatin OR Cerivastatin OR "PCSK9 inhibitor*" OR "PCSK9" OR Evolocumab OR Alirocumab OR Inclisiran OR "Fibric acid*" OR "Fibrate*" OR Fenofibric OR Fenofibrate OR gemfibrozil OR "Bile acid sequestrant*" OR "bile acid resin*" OR Colesevelam OR Cholestyramine OR Colestipol OR "Nicotinic acid*" OR "Niacin" OR Nicotinic OR "pyridine-3-carboxylic acid" OR nicotinamide OR niacinamide OR niacin OR "pyridine-3-carboxamide" OR "vitamin B3" OR "Cholesterol absorption inhibitor*" OR Ezetimibe OR "Omega-3 fatty acid*" OR "Omega-3" OR "omega-3-acid" OR Lovaza OR "Adenosine triphosphate citrate synthase inhibitor*" OR "ATP citrate synthase inhibitor*" OR "Adenosine triphosphate-citrate synthase inhibitor*" OR "ATP-citrate synthase inhibitor*" OR "Adenosine triphosphate citrate-synthase inhibitor*" OR "ATP citrate-synthase inhibitor*" OR "ATP citrate lyase" OR Bempedoic)) | 453,038 |
| #2 | (TITLE-ABS-KEY("COVID-19" OR "coronavirus" OR "SARS-CoV-2" OR "severe acute respiratory syndrome")) | 524,702 |
| #3 | (TITLE-ABS-KEY(trial OR random* OR intervention* OR placebo)) | 6,232,426 |
| **#4** | **#1 AND #2 AND #3** | **742** |
| ***Embase*** | | |
| #1 | (("Statin*" OR Atorvastatin OR Fluvastatin OR Lovastatin OR Pitavastatin OR Pravastatin OR Rosuvastatin OR Simvastatin OR Cerivastatin OR "PCSK9 inhibitor*" OR "PCSK9" OR Evolocumab OR Alirocumab OR Inclisiran OR "Fibric acid*" OR "Fibrate*" OR Fenofibric OR Fenofibrate OR gemfibrozil OR "Bile acid sequestrant*" OR "bile acid resin*" OR Colesevelam OR Cholestyramine OR Colestipol OR "Nicotinic acid*" OR "Niacin" OR Nicotinic OR "pyridine-3-carboxylic acid" OR nicotinamide OR niacinamide OR niacin OR "pyridine-3-carboxamide" OR "vitamin B3" OR "Cholesterol absorption inhibitor*" OR Ezetimibe OR "Omega-3 fatty acid*" OR "Omega-3" OR "omega-3-acid" OR Lovaza OR "Adenosine triphosphate citrate synthase inhibitor*" OR "ATP citrate synthase inhibitor*" OR "Adenosine triphosphate-citrate synthase inhibitor*" OR "ATP-citrate synthase inhibitor*" OR "Adenosine triphosphate citrate-synthase inhibitor*" OR "ATP citrate-synthase inhibitor*" OR "ATP citrate lyase" OR Bempedoic):ti,ab,kw) | 221,458 |
| #2 | (("COVID-19" OR "coronavirus" OR "SARS-CoV-2" OR "severe acute respiratory syndrome"):ti,ab,kw) | 385,630 |
| #3 | ((trial OR random* OR intervention* OR placebo):ti,ab,kw) | 3,928,681 |
| **#4** | **#1 AND #2 AND #3** | **279** |
| ***Web of Science*** | | |
| #1 | (TS=("Statin*" OR Atorvastatin OR Fluvastatin OR Lovastatin OR Pitavastatin OR Pravastatin OR Rosuvastatin OR Simvastatin OR Cerivastatin OR "PCSK9 inhibitor*" OR "PCSK9" OR Evolocumab OR Alirocumab OR Inclisiran OR "Fibric acid*" OR "Fibrate*" OR Fenofibric OR Fenofibrate OR gemfibrozil OR "Bile acid sequestrant*" OR "bile acid resin*" OR Colesevelam OR Cholestyramine OR Colestipol OR "Nicotinic acid*" OR "Niacin" OR Nicotinic OR "pyridine-3-carboxylic acid" OR nicotinamide OR niacinamide OR niacin OR "pyridine-3-carboxamide" OR "vitamin B3" OR "Cholesterol absorption inhibitor*" OR Ezetimibe OR "Omega-3 fatty acid*" OR "Omega-3" OR "omega-3-acid" OR Lovaza OR "Adenosine triphosphate citrate synthase inhibitor*" OR "ATP citrate synthase inhibitor*" OR "Adenosine triphosphate-citrate synthase inhibitor*" OR "ATP-citrate synthase inhibitor*" OR "Adenosine triphosphate citrate-synthase inhibitor*" OR "ATP citrate-synthase inhibitor*" OR "ATP citrate lyase" OR Bempedoic)) | 207,834 |
| #2 | (TS=("COVID-19" OR "coronavirus" OR "SARS-CoV-2" OR "severe acute respiratory syndrome")) | 347,652 |
| #3 | (TS=(trial OR random* OR intervention* OR placebo)) | 4,055,767 |
| **#4** | **#1 AND #2 AND #3** | **247** |
| ***Total*** | | ***1,607*** |
| ***Total without duplicates*** | | ***1,035*** |

**Previous meta-analyses on the effects of statin on COVID-19 outcomes**

*1. Lao US, Law CF, Baptista-Hon DT, Tomlinson B. Systematic Review and Meta-Analysis of Statin Use and Mortality, Intensive Care Unit Admission and Requirement for Mechanical Ventilation in COVID-19 Patients. J Clin Med. 2022;11(18).*

*2. Vahedian-Azimi A, Mohammadi SM, Banach M, Beni FH, Guest PC, Al-Rasadi K, et al. Improved COVID-19 Outcomes following Statin Therapy: An Updated Systematic Review and Meta-analysis. Biomed Res Int. 2021;2021:1901772.*

*3. Diaz-Arocutipa C, Melgar-Talavera B, Alvarado-Yarasca Á, Saravia-Bartra MM, Cazorla P, Belzusarri I, et al. Statins reduce mortality in patients with COVID-19: an updated meta-analysis of 147 824 patients. Int J Infect Dis. 2021;110:374-81.*

*4. Kow CS, Hasan SS. The Association Between the Use of Statins and Clinical Outcomes in Patients with COVID-19: A Systematic Review and Meta-analysis. Am J Cardiovasc Drugs. 2022;22(2):167-81.*

*5. Yetmar ZA, Chesdachai S, Kashour T, Riaz M, Gerberi DJ, Badley AD, et al. Prior Statin Use and Risk of Mortality and Severe Disease From Coronavirus Disease 2019: A Systematic Review and Meta-analysis. Open Forum Infect Dis. 2021;8(7):ofab284.*

*6. Kollias A, Kyriakoulis KG, Kyriakoulis IG, Nitsotolis T, Poulakou G, Stergiou GS, et al. Statin use and mortality in COVID-19 patients: Updated systematic review and meta-analysis. Atherosclerosis. 2021;330:114-21.*

*7. Zein A, Sulistiyana CS, Khasanah U, Wibowo A, Lim MA, Pranata R. Statin and mortality in COVID-19: a systematic review and meta-analysis of pooled adjusted effect estimates from propensity-matched cohorts. Postgrad Med J. 2022;98(1161):503-8.*

*8. Chow R, Im J, Chiu N, Chiu L, Aggarwal R, Lee J, et al. The protective association between statins use and adverse outcomes among COVID-19 patients: A systematic review and meta-analysis. PLoS One. 2021;16(6):e0253576.*

*9. Wu KS, Lin PC, Chen YS, Pan TC, Tang PL. The use of statins was associated with reduced COVID-19 mortality: a systematic review and meta-analysis. Ann Med. 2021;53(1):874-84.*

*10. Vahedian-Azimi A, Mohammadi SM, Heidari Beni F, Banach M, Guest PC, Jamialahmadi T, et al. Improved COVID-19 ICU admission and mortality outcomes following treatment with statins: a systematic review and meta-analysis. Arch Med Sci. 2021;17(3):579-95.*

*11. Hariyanto TI, Kurniawan A. Statin and outcomes of coronavirus disease 2019 (COVID-19): A systematic review, meta-analysis, and meta-regression. Nutr Metab Cardiovasc Dis. 2021;31(6):1662-70.*

*12. Permana H, Huang I, Purwiga A, Kusumawardhani NY, Sihite TA, Martanto E, et al. In-hospital use of statins is associated with a reduced risk of mortality in coronavirus-2019 (COVID-19): systematic review and meta-analysis. Pharmacol Rep. 2021;73(3):769-80.*

*13. Pal R, Banerjee M, Yadav U, Bhattacharjee S. Statin use and clinical outcomes in patients with COVID-19: An updated systematic review and meta-analysis. Postgrad Med J. 2022;98(1159):354-9.*

*14. Hariyanto TI, Kurniawan A. Statin therapy did not improve the in-hospital outcome of coronavirus disease 2019 (COVID-19) infection. Diabetes Metab Syndr. 2020;14(6):1613-5.*
